# Low red to far-red light ratio promotes salt tolerance by improving leaf photosynthetic capacity in cucumber

**DOI:** 10.3389/fpls.2022.1053780

**Published:** 2023-01-06

**Authors:** Yanxiu Miao, Xingxing Gao, Bin Li, Wenjiao Wang, Longqiang Bai

**Affiliations:** ^1^ College of Horticulture, Shanxi Agricultural University, Jinzhong, China; ^2^ Collaborative Innovation Center for Improving Quality and Increase profits of Protected Vegetables in Shanxi, Shanxi Agricultural University, Jinzhong, China

**Keywords:** red to far-red light ratio, salt stress, cucumber, photosynthetic electron transport, Calvin cycle

## Abstract

Soil salinity severely inhibits leaf photosynthesis and limits agricultural production. Red to far-red light ratio (R/FR) affects leaf photosynthesis under salt stress, however, its regulation mechanism is still largely unknown. This study investigated the effects of different R/FR on plant growth, gas exchange parameters, photosynthetic electron transport, Calvin cycle and key gene expression under salt stress. Cucumber seedlings were exposed to four treatments including 0 mM NaCl and R/FR=7 (L7, control), 0 mM NaCl and R/FR=0.7 (L0.7), 80 mM NaCl and R/FR=7 (H7) and 80 mM NaCl and R/FR=0.7 (H0.7) for 9 days in an artificial climate chamber. The results showed that compared to L7 treatment, H7 treatment significantly reduced relative growth rate (RGR), CO_2_ assimilation rate (*P*_n_), maximum photochemical efficiency PSII (*F*_v_/*F*_m_), most JIP-test parameters and total Rubisco activity, indicating that salt stress severely inhibited photosynthetic electron transport from PSII to PSI and blocked Calvin cycle in cucumber leaves. However, these suppressions were effectively alleviated by low R/FR addition (H0.7 treatment). Compared to H7 treatment, H0.7 treatment significantly increased RGR and *P*_n_ by 209.09% and 7.59%, respectively, enhanced *F*_v_/*F*_m_, maximum quantum yield for primary photochemistry (*φ*_Po_), quantum yield for electron transport (*φ*_Eo_) and total Rubisco activity by 192.31%, 17.6%, 36.84% and 37.08%, respectively, and largely up-regulated expressions of most key genes involved in electron transport and Calvin cycle. In conclusion, low R/FR effectively alleviated the negative effects of salt stress on leaf photosynthesis by accelerating photosynthetic electron transport from PSII to PQ pool and promoting Calvin cycle in cucumber plants. It provides a novel environmentally friendly light-quality regulation technology for high efficiency salt-resistant vegetable production.

## Introduction

Soil salinity, a global environmental problem, occurs in approximately 7% of the world’s total land area and 20% of irrigated land (Shabala and Cuin, 2008). Salt stress not only negatively affects plant growth and production, but also induces a series of physiological and metabolic disorders, especially photosynthesis (Kalaji et al., 2011; Zhang J. et al., 2016). Salt stress-induced osmotic stress decreases water absorption in root, causes water loss in leaves, and thus plays negative roles in leaf photosynthesis. Salt stress also stimulates synthesis of reactive oxygen species (ROS), which seriously destructs photosynthetic organs and components, and inhibits leaf photosynthetic characteristics, such as CO_2_ assimilation rate, stomatal conductance, maximum photochemical efficiency PSII (*F*_v_/*F*_m_) (Ma et al., 2017; Niu et al., 2019; Gong et al., 2020). Therefore, it is necessary to improve salt tolerance by promoting leaf photosynthetic capacity.

Light quality participants in the regulation of growth, physiological and yield characteristics in vegetable crops. Red to far-red light ratio (R/FR), one of the important light environment factors, participants in seed germination, plant photomorphogenesis, physiological metabolism and gene expression (Demotes-Mainard et al., 2015; Holalu and Finlayson, 2017). R/FR is about 7 under light-emitting diode (LED) lamp, 1.14 under sunny condition and 0.09-0.7 under shade condition. Low R/FR induces shade avoidance syndrome (SAS) responses, such as increased internode, petiole, stem, leaf length and plant dry weight, apical dominance and early flowering (Franklin, 2008). Low R/FR also takes parts in several physiological changes, especially in leaf photosynthetic characteristics, it dramatically increases leaf net photosynthetic rate and effective photochemical quantum yield of PSII (Φ_II_), accelerates cyclic electron transport around PSI, but decreases leaf chlorophyll *a*/*b* in horticultural crops, such as tomato, soybean and lettuce (Zhen and Iersel, 2017; Kalaitzoglou et al., 2019; Yang et al., 2020).

R/FR not only influences plant growth and physiological metabolism, but also effectively alleviates injuries caused by abiotic stresses, including salt, cold and drought stresses and so on (Courbier and Pierik, 2019; Ahres et al., 2020; Gyugos et al., 2021). Under salt stress, low R/FR increased the stability of Phytochrome interaction factor (PIF) and upregulated brassinosteroid and auxin signaling, thus promoted hypocotyl growth in Arabidopsis (Hayes et al., 2019). Low R/FR also up-regulated *SODCC.2*, *GPX1*, *APX2* and *CAT1* gene expressions and increased antioxidant enzyme (e.g., SOD, POD and CAT) activities, thus enhanced salt resistance in tomato plants (Cao et al., 2018; Wang Y. et al., 2021). Cockburn et al. (1996) found that under salt stress, low R/FR enhanced PEP carboxylase activity, and caused accumulation of CAM isoform of PEP carboxylase isozyme and increased terpineol and soluble carbohydrate contents, finally improved salt tolerance in *Mesembryanthemum crystallinum* plant. However, these researches on salt tolerance of R/FR basically focus on plant growth and antioxidant capacity, little is known about how R/FR regulates leaf photosynthetic responses to salt stress. It is of great research significance to explore the regulation roles of R/FR on specific processes of photosynthetic electron transport chain and Calvin cycle simultaneously under salt stress.

Cucumber (*Cucumis sativus* L.) is an important worldwide economic vegetable crop, and its growth and production are severely limited by salt stress (Miao et al., 2020; Wang W. et al., 2021). Cucumber is sensitive to light quality, and its photomorphogenesis and photosynthetic characteristics are easily regulated by R/FR (Shibuya et al., 2015; Miao et al., 2019; Jeong et al., 2020). In the present study, we investigated the effect of R/FR on leaf gas exchange parameters, photosynthetic electron transfer capacity, Calvin cycle and key gene expression in salt-stressed cucumber plants. According to the regulation mechanism of R/FR on leaf photosynthesis under salt stress, this study will provide a more theoretical basic and new light quality control method to improve salt tolerance in cucumber production.

## Materials and methods

### Plant materials and growth conditions

**Figure 1:**
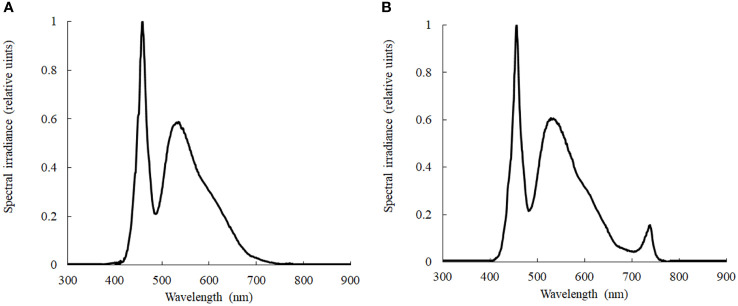
Relative spectral distribution in different light treatments. **(A)**, R/FR=7; **(B)**, R/FR=0.7.

Cucumber (*Cucumis sativus* L.cv. ‘Jinchun 4’) seeds were germinated at 28°C, then sowed into a hydroponic tank in an artificial climate chamber. After the second true leaf has fully developed, all plants were exposed to four treatments, including 0 mM NaCl and R/FR=7 (L7, control), 0 mM NaCl and R/FR=0.7 (L0.7), 80 mM NaCl and R/FR=7 (H7) and 80 mM NaCl and R/FR=0.7 (H0.7). The hydroponic tank was filled with full strength Hoagland nutrient solution contained 0 mM or 80 mM NaCl, respectively. Red and far-red light were provided by LED lamps with maximum intensity at 660nm and 730nm, respectively. Red to far-red light ratio (R/FR) was calculated from photon irradiance for the bands 655-656 nm and 725-735nm according to Kotilainen et al. (2020). The light intensity and spectral distribution were determined by Avaspec-2048 fiber optic spectrometer (AVANTES, Netherlands) in the range 400-950nm with a spectral resolution of 1 nm (**Figure 1**). The day/night temperature, light intensity, photoperiod and relative humidity were 26°C/18°C, 250μmol·m^2^·s^−1^, 12h·d^-1^ and 60%-80%, respectively. The cucumber samples were harvested at day 9 after treatments and the experiments were repeated at least three times.

### Plant growth parameters

Four cucumber seedlings from each treatment were harvested on days 0 and 9 after treatments. Leaf area was measured using a LI-3000C portable leaf area meter (LI-COR, USA) and dry weight was fully dried at 80°C in an oven for 2 days. Relative growth rate (RGR), net assimilation rate (NAR), leaf area ratio (LAR), leaf dry weight ratio (LWR), and specific leaf area (SLA) were calculated according to Hunt (1978).

### Leaf photosynthetic pigment and carbohydrate contents

The samples from second leaves were harvested to measure photosynthetic pigment and carbohydrate contents. The photosynthetic pigments, soluble sugar and starch contents was measured using the method of Zhao X. et al. (2022), fructose and sucrose content was determined by resorcinol spectrophotometry according to Gao (2006). Four cucumber seedlings were selected for each treatment.

### Gas exchange parameters

Gas exchange parameters were determined on the fully expanded second leaves at a similar position. Cucumber leaves exposed to different R/FR were measured under their own light quality conditions with a LI-6800 gas exchange analyzer and a standard transparent leaf chamber (LI-COR, USA). When leaves were clamped and reached steady-state condition in leaf chamber, gas exchange parameters such as CO_2_ assimilation rate (*P*_n_), stomatal conductance (*g*_sw_), intercellular CO_2_ concentration (*C*_i_) and transpiration rate (*T*_r_) were measured. The light intensity was 250 μmol·m^-2^·s^-1^, leaf temperature was 26°C, relative humidity was 60%-70%, CO_2_ concentration was 400 μmol·mol^-1^ in leaf chamber. Four cucumber seedlings were selected for each treatment.

The CO_2_ response curve was measured with LI-6800 gas exchange analyzer using the same gas exchange parameters as described above. According to the procedure described by Long Bernacchi (2003), light intensity was 1500 μmol·m^-2^·s^-1^, CO_2_ concentration in leaf chamber was initially 400 μmol·mol^-1^ for 5 min, then followed by 400, 300, 250, 200, 150, 100, 50, 400, 600, 800, 1000, 1200, 1500 and 1800 μmol·mol^-1^. Three cucumber seedlings were selected for each treatment.

### Chlorophyll fluorescence parameters

The maximum photochemical efficiency PSII (*F*_v_/*F*_m_) image was measured on the second leaves using a Maxi Imaging-pam fluorescence system (Walz, Germany). Details of procedure and measurement system were described in Perreault et al. (2010). After 30 min of dark adaptation, minimum fluorescence (*F*_o_) was measured with a modulated light, then maximum fluorescence (*F*_m_) was determined with a saturation pulse. *F*_v_/*F*_m_ was calculated automatically using *F*_v_/*F*_m_ =(*F*_m_-*F*_o_)/*F*_m_.

The light induction transient of chlorophyll fluorescence (OJIP) curves were determined with a FluorPen FP 110 handheld chlorophyll fluorometer (Photon systems instruments, Czech Republic). After 30 min of dark adaptation, OJIP curve was induced by pulsed light of 3000 μmol·m^-2^·s^-1^. The relative variable fluorescence (*V*_t_) was calculated as *V*_t_=(*F*_t_−*F*_o_)/(*F*_p_−*F*_o_) according to Suzuki et al. (2011). Where, *V*_t_ and *F*_t_ represent relative variable fluorescence and fluorescence intensity at time t, respectively, *F*_o_ and *F*_p_ represent initial and maximum fluorescence intensity, respectively. JIP-test parameters were calculated according to Strasser et al. (2004). The formulas are as follows: Maximum quantum yield for primary photochemistry, *φ*_Po_=TR_o_/ABS=[1-(*F*_o_/*F*_m_)], quantum yield for electron transport, *φ*_Eo_=ET_o_/ABS=[1-(*F*_o_-*F*_m_)](1-*V*_J_), quantum yield for reduction of end electron acceptors at the PSI acceptor side, *φ*_Ro_=RE_o_/ABS=[1-(*F*_o_/*F*_m_)](1-*V*_J_), efficiency/probability that an electron moves further than Q_A_^-^, *Ψ*_Eo_=ET_o_/TR_o_=1-*V*_J_, efficiency/probability with which an electron from the intersystem electron carriers is transferred to reduce end electron acceptors at the PSI acceptor side, *δ*_Ro_=RE_o_/ET_o_=(1-*V*_I_)/(1-*V*_J_), Q_A_ reducing RCs per PSII antenna Chl, RC/ABS=*φ*_Po_(*V*_J_/*M*_o_), performance index (potential) for energy conservation from photons absorbed by PSII to the reduction of intersystem electron acceptors, PI_ABS_=RC/ABS×[*φ*_Po_/(1-φ_Po_)]×[*Ψ*_Eo_/(1-*Ψ*_Eo_)], performance index (potential) for energy conservation from photons absorbed by PSII to the reduction of PSI end acceptors, PI_total_=PI_ABS_·*δ*_Ro_/(1-*δ*_Ro_). Four cucumber seedlings were selected for each treatment.

### Rubisco activity

The second leaves of four plants from each treatment were selected and snap frozen in liquid nitrogen, then stored at -80°C in a refrigerator. The initial and total Ribulose 1,5-bisphosphate carboxylase/oxygenase (Rubisco) activity and protein content were determined and calculated according to Zhang et al. (2013) and Wang et al. (2016).

### Quantitative real-time PCR analysis

Total RNA was extracted from the second leaves using TRIZOL reagent (Shanghai Blue Quarter Technology Development Co., Ltd., China) according to the method of Wang et al. (2009). Reverse transcription was performed with a CjamQTM Universal SYBR^®^ qPCR Master Mix kit (Vazyme Biotech Co., Ltd, China) and real-time PCR (qPCR) was performed using a HiScript^®^ III RT SuperMix kit (Vazyme Biotech Co., Ltd, China). The previously published primer sequences according to Miao et al. (2016) and Zhao H. et al. (2022) were used in this study (**Table 1**). Three cucumber seedlings were selected for each treatment.

**Table 1:** Primers used for qPCR.

Gene name	Gene ID	Forward primer	Reverse primer
***Actin***	Csa6G484600.2	5’-ATGGCCGATGCCGAGGATAT-3’	5’-TAGGAGCATCATCACCAGCAAAAC-3’
***psb*A**	CsaUNG024230.1	5’-GTATTCCAGGCTGAGCACAACATC-3’	5’-TACCTAAAGCGGTGGACCAGATAC-3’
***psb*B**	Csa5G589930.1	5’-GGTATTTGGAGTTACGAAGGTGTG-3’	5’-CCCAACCCTGAGAGAAATAAATGA-3’
***psa*A**	Csa3G895840.1	5’-GATTTCTCATAGTTGGTGCTGCTG-3’	5’-TACAAACCAAAACTGTGAAAGCCT-3’
***psa*B**	CsaV3_UNG201530.1	5’-ATTTGGACATCTTGTTTGGGCTAC-3’	5’-TGATGTAGAGGCAATCAAGAAAGC-3’
***rbcL***	CsaV3_UNG203690.1	5’-TACTGATATCTTGGCAGCATTCCG-3’	5’-AAGATTCAGCAGCTACAGCGGC-3’
***rbcS***	Csa5G609710.1	5’-ATGGCTTCATCCATTCTCTCATCC-3’	5’-CCAGTGAATGGTGCTACCATGCTA-3’
***Rca***	Csa6G188680.2	5’-GAATATGGCAACATGCTCGTCATG-3’	5’-TCCAAGAGCAGCTTCGTTCAGAT-3’
***TPI***	Csa2G263900.1	5’-CTCTCTTTCACAACGTCCACTCACA-3’	5’-ACCAACGAAGAACTTGCCGGAG-3’
***FBPase***	Csa4G307350.1	5’-AATCTCTCGTTCTCTCTCTCGCCTC-3’	5’-CGCTATGCCTTCTATTTCCACGG-3’
***SBPase***	Csa5G198220.1	5’-CAGTGTCCTCCTCATACTTGGGTTG-3’	5’-CTGGGAAGAAAGATTGGGGAGAAA-3’
***Rupe***	Csa1G038360.2	5’-TCCCAAGTCAGTGGGTTTATCGGAG-3’	5’-AACCTTCTCCTCGAAACGGTAAGAG-3’
***PRK***	Csa3G638540.1	5’-ATCCACACCCTCATTCATTTCTCC-3’	5’-GCAGTTGAGGGAGTGAAGAAGAAGA-3’

### Statistical analysis

All statistical analysis was carried out with SPSS 21.0 software (IBM Corporation, USA). The data were analyzed by one-way analysis of variance (ANOVA) and significant differences among means were assessed by Duncan’s test (P <0.05).

## Results

### Plant growth analysis

**Figure 2:**
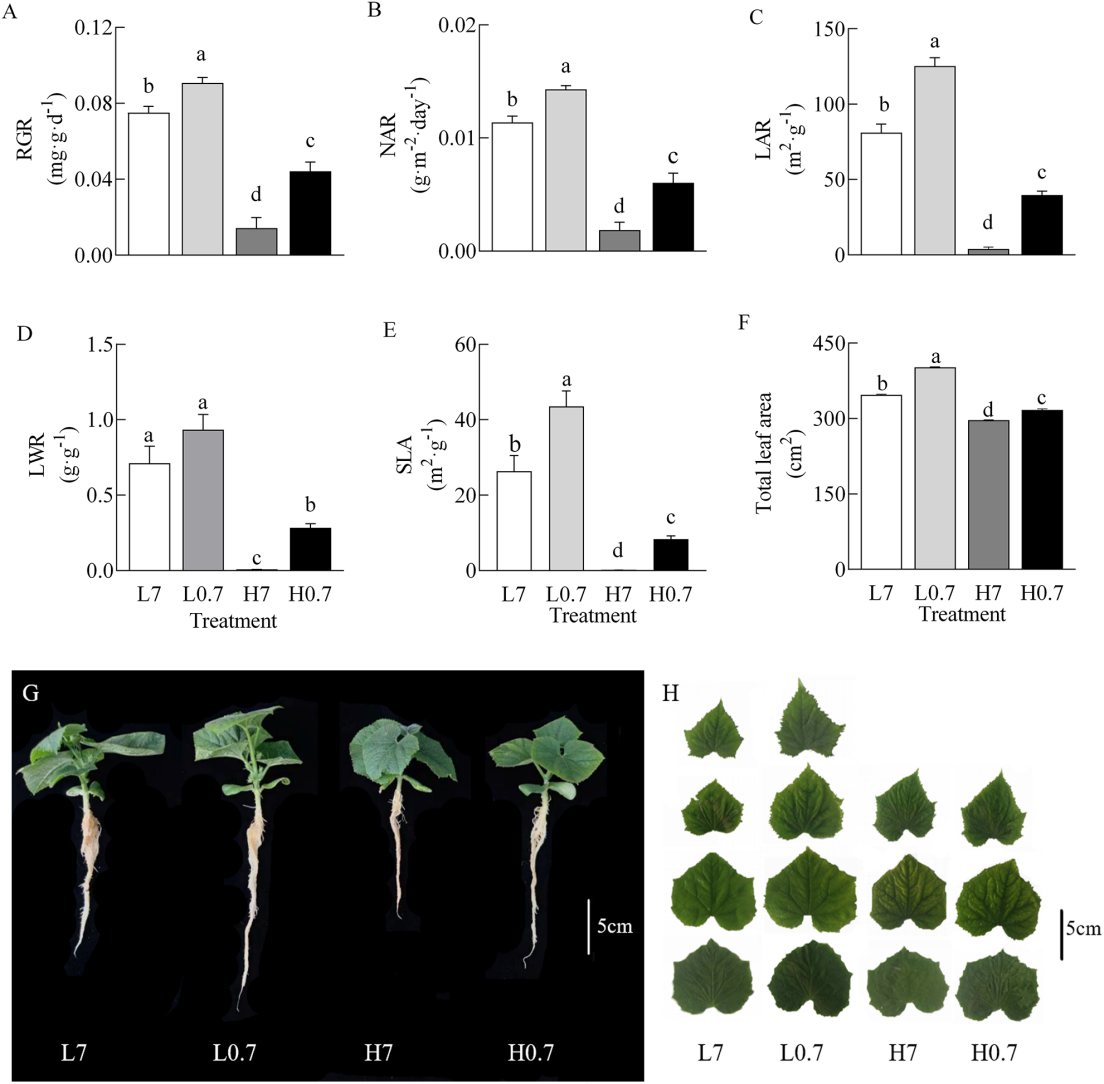
Effect of R/FR on growth parameters of cucumber seedlings under salt stress. **(A)**, RGR, relative growth rate; **(B)**, NAR, net assimilation rate; **(C)**, LAR, leaf area ratio; **(D)**, LWR, leaf dry weight ratio; **(E)**, SLA, specific leaf area; **(F)**, total leaf area; Cucumber seedlings **(G)** and total true leaves **(H)** on day 9 after treatment. L7, 0 mM NaCl and R/FR=7; L0.7, 0 mM NaCl and R/FR=0.7; H7, 80 mM NaCl and R/FR=7; H0.7, 80 mM NaCl and R/FR=0.7. Different letters indicate significant differences (P< 0.05; n= 4).

Generally, compared to L7 treatment, most plant parameters (e.g., Relative growth rate (RGR), net assimilation rate (NAR), leaf area ratio (LAR), specific leaf area (SLA) and total leaf area) were significantly increased by L0.7 treatment, while all plant parameters were significantly decreased by H7 and H0.7 treatments (**Figure 2**). Most plant parameters (e.g., RGR, NAR, LAR, LWR and total leaf area) were significantly higher for H0.7 treatment than for H7 treatment. These results indicated that salt stress seriously inhibited plant growth, however, the negative effects of salt stress on plant growth were effectively alleviated by low R/FR.

### Leaf photosynthetic pigment and carbohydrate

Compared to L7 treatment, chlorophyll *a*, chlorophyll *b*, soluble sugar and starch contents were significantly increased by L0.7 treatment, however, all photosynthetic pigment and carbohydrate contents were significantly decreased by H7 treatment, chlorophyll *a*, soluble sugar, fructose and sucrose contents were significantly reduced by H0.7 treatments (**Table 2**). The chlorophyll *a* and all carbohydrate (e.g., soluble sugar, fructose, sucrose and starch) contents were significantly higher for H0.7 treatment than for H7 treatment. This indicated that the negative effects of salt stress on photosynthetic pigment and carbohydrate were dramatically reduced by low R/FR.

**Table 2:** Effect of R/FR ratio on photosynthetic pigment and carbohydrate contents in cucumber leaves under salt stress.

Treatment	Chlorophyll *a* content (mg·g^-1^ FW)	Chlorophyll *b* content (mg·g^-1^ FW)	Carotenoid content (mg·g^-1^ FW)	Soluble sugar content (mg·g^-1^ FW)	Fructose content (mg·g^-1^ FW)	Sucrose content(mg·g^-1^ FW)	Starch content(mg·g^-1^ FW)
L7	0.45 ± 0.02 b	0.14 ± 0.01 b	0.09 ± 0.00 ab	2.25 ± 0.09 b	3.89 ± 0.04 a	3.49 ± 0.04 a	0.14 ± 0.01 b
L0.7	0.65 ± 0.01 a	0.16 ± 0.01 a	0.10 ± 0.01 a	2.55 ± 0.03 a	3.96 ± 0.09 a	3.54 ± 0.02 a	0.30 ± 0.03 a
H7	0.31 ± 0.01 d	0.11 ± 0.00 c	0.08 ± 0.00 c	0.50 ± 0.04 d	2.6 ± 0.08 c	2.71 ± 0.05 c	0.08 ± 0.01 c
H0.7	0.37 ± 0.02 c	0.12 ± 0.01 bc	0.09 ± 0.01 bc	1.75 ± 0.05 c	1.75 ± 0.05 b	3.33 ± 0.07 b	0.14 ± 0.01 b

L7, 0 mM NaCl and R/FR=7; L0.7, 0 mM NaCl and R/FR=0.7; H7, 80 mM NaCl and R/FR=7; H0.7, 80 mM NaCl and R/FR=0.7. Different letters within the same column represent significant differences (P<0.05, n=4).

### Leaf gas exchange parameter

**Figure 3:**
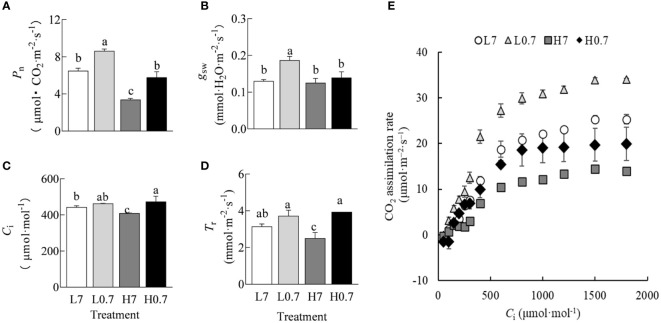
Effect of R/FR on gas exchange parameters in cucumber leaves under salt stress. **(A)**, *P*_n_, CO_2_ assimilation rate; **(B)**, *g*_sw_, stomatal conductance; **(C)**, *C*_i_, intercellular CO_2_ concentration; **(D)**, *T*_r_, transpiration rate; **(E)**, CO_2_ response curve. All data in **(A–E)** were reported as the arithmetic mean ± standard error (n=4 and n=3, respectively).

Compared to L7 treatment, *P*_n_ and *g*_sw_ were significantly increased by L0.7 treatment, *C*_i_ was significantly enhanced by H0.7 treatment, while most gas exchange parameters (e.g., *P*_n_, *C*_i_ and *T*_r_) were significantly reduced by H7 treatment (**Figure 3A–D**). Compared to H7 treatment, *P*_n_ and *T*_r_ were significantly increased by H0.7 treatment.

For all treatments, CO_2_ assimilation rate increased quickly with increasing *C*_i_ and reached maximum values when *C*_i_ was above 1000 μmol·mol^-1^ (**Figure 3E**). It was obvious that L0.7 treatment had the highest CO_2_ assimilation rate, followed by L7, H0.7 and H7 treatments.

### Leaf chlorophyll fluorescence parameter

**Figure 4:**
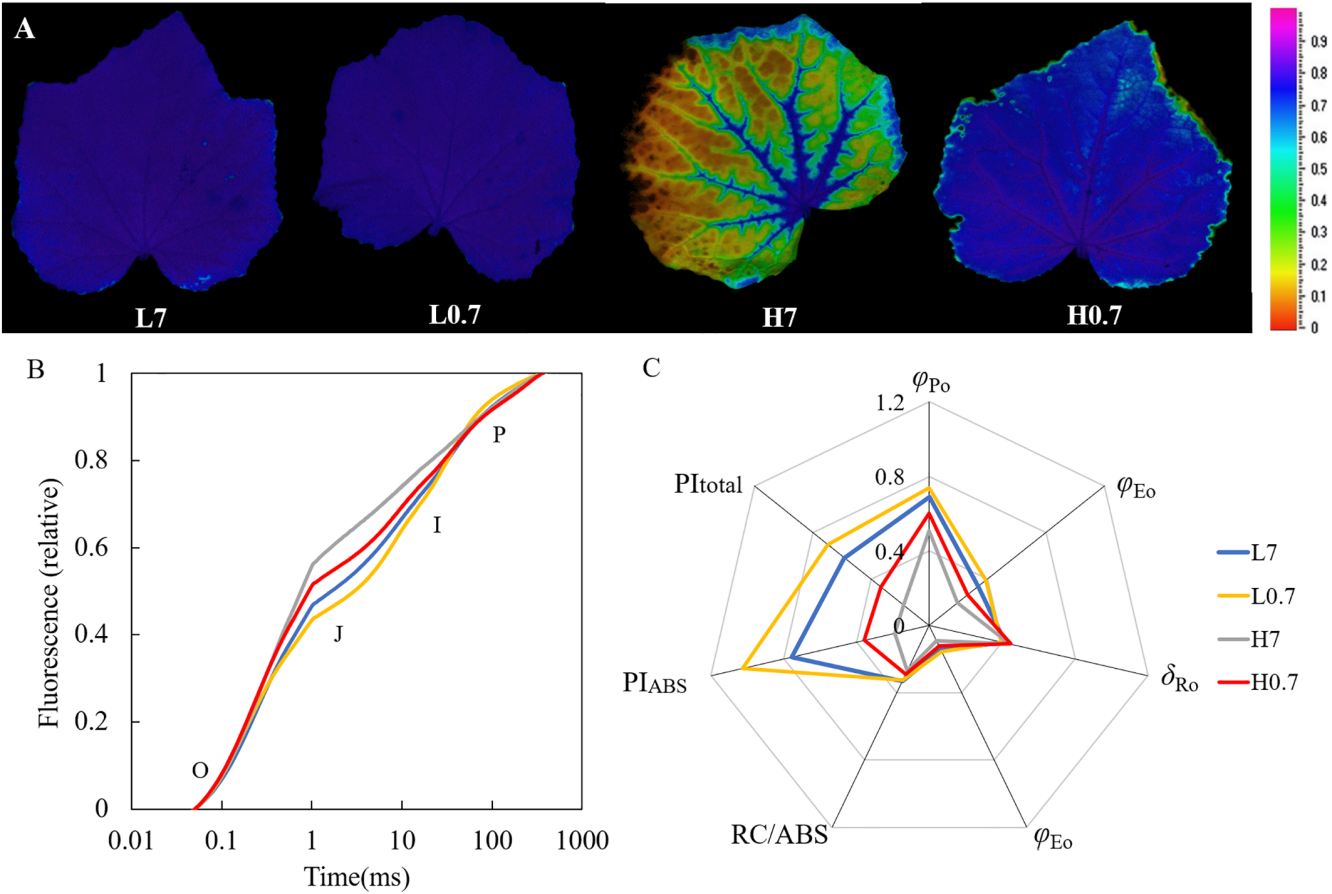
Effect of R/FR on chlorophyll fluorescence parameters in cucumber leaves under salt stress. **(A)**, maximal photochemical quantum yield of PSII (*F*_v_/*F*_m_) images of cucumber leaves; **(B)**, Chlorophyll *a* fluorescence transient (OJIP) curve; **(C)**, JIP-test parameters calculated according to OJIP curve. Different letters indicate significant differences (P< 0.05; n= 4).

The cucumber leaves in L7 and L0.7 treatments showed a homogeneous *F*_v_/*F*_m_ distribution centred around an *F*_v_/*F*_m_ of 0.80, while leaf in H7 treatment had a heterogeneous distribution with a high *F*_v_/*F*_m_ around the veins and an extremely low level of *F*_v_/*F*_m_ (0.26) between the veins, *F*_v_/*F*_m_ distribution in H0.7 treatment was relative homogeneous around an *F*_v_/*F*_m_ of 0.77 (**Figure 4A** and **Table S1**).

The typical polyphasic OJIP rise was found in L7 treatment (**Figure 4B**). The J (*V*_J_, at 2ms) step was significantly higher for L0.7 treatment than for L7 treatment and was significantly higher for H0.7 treatment than for H7 treatment (**Table S1**). Compared to L7 treatment, *φ*_Eo_ and PI_ABS_ were significantly increased by L0.7 treatment, however, most JIP-test parameters (e.g., *φ*_Po_, *φ*_Eo_, *φ*_Ro_, RC/ABS, PI_ABS_ and PI_total_) were reduced by H7 treatment, and *φ*_Po_, *φ*_Eo_ and PI_ABS_ were significantly decreased by H0.7 treatment (**Figure 4C** and **Table S1**). Compared with H7 treatment, *φ*_Po_ and *φ*_Eo_ were significantly increased by H0.7 treatment.

### Rubisco activity

**Figure 5:**
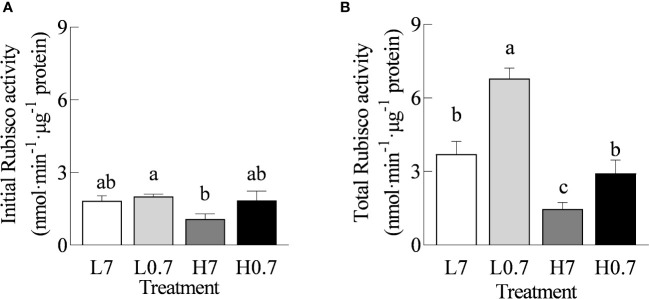
Effect of R/FR on Rubisco activity in cucumber leaves under salt stress. **(A)**, Initial Rubisco activity; **(B)**, Total Rubisco activity. Different letters indicate significant differences (P< 0.05; n= 4).

Compared with L7 treatment, total Rubisco activity was statistically increased by L0.7 treatment, while total Rubisco activity was significantly reduced by H7 treatments (**Figure 5**). Total Rubisco activity was much higher for H0.7 treatment than for H7 treatment.

### Expression levels of key genes involved in electron transport and Calvin cycle

**Figure 6:**
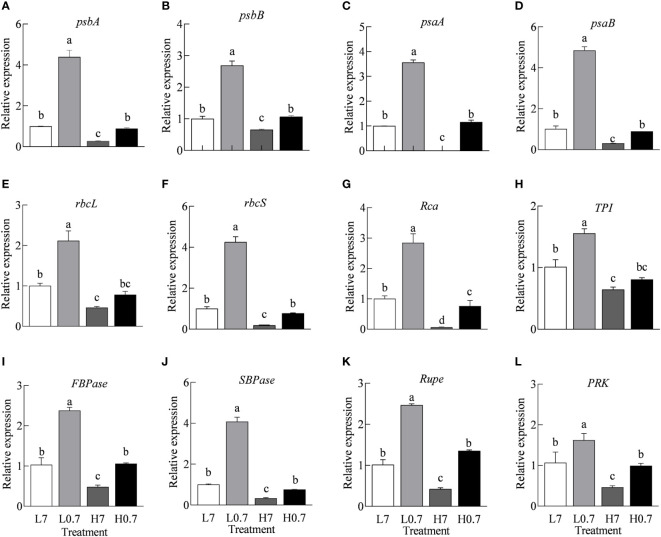
Effect of R/FR on relative expression levels of key genes involved in electron transport and Calvin cycle in cucumber leaves under salt stress. The relative expression levels of psbA **(A)**, psbB **(B)**, psaA **(C)** and psaB **(D)** genes involved in electron transport and relative expression levels of rbcL **(E)**, rbcS **(F)**, Rca **(G)**, TPI **(H)**, FBPase **(I)**, SBPase **(J)**, Rupe **(K)** and PRK **(L)** genes involved in Calvin cycle *rbcL*, ribulose-1,5-bisphosphate carboxylase/oxygenase large subunit; *rbcS*, ribulose-1,5-bisphosphate carboxylase/oxygenase small subunit; *Rca*, ribulose-1,5-bisphosphate carboxylase/oxygenase activase; *TPI*, propanosyl phosphate isomerase; *FBPase*, fructose-1,6-bisphosphatase; *SBPase*, scenic heptulose-1,7-bisphosphatase; *Rupe*, ribulose-5-phosphate epimerase; *PRK*, ribulose-5-phosphate kinase. Different letters indicate significant differences (P< 0.05; n= 3).

In general, compared to L7 treatment, expression levels of all key genes (e.g., *psbA*, *psbB*, *psaA*, *psaB*, *rbcL*, *rbcS*, *Rca*, *TPI*, *FBPase*, *SBPase*, *Rupe* and *PRK*) involved in photosynthetic electron transport and Calvin cycle were significantly up-regulated by L0.7 treatment, however, all gene expressions were obviously down-regulated by H7 treatment, only *Rca* gene expression was down-regulated by H0.7 treatments when compared to L7 treatment (**Figure 6**). The transcription levels of all genes except for *rbcL* and *TPI* were much higher in H0.7 treatment than H7 treatment.

### R/FR affects leaf photosynthesis under salt stress

**Figure 7:**
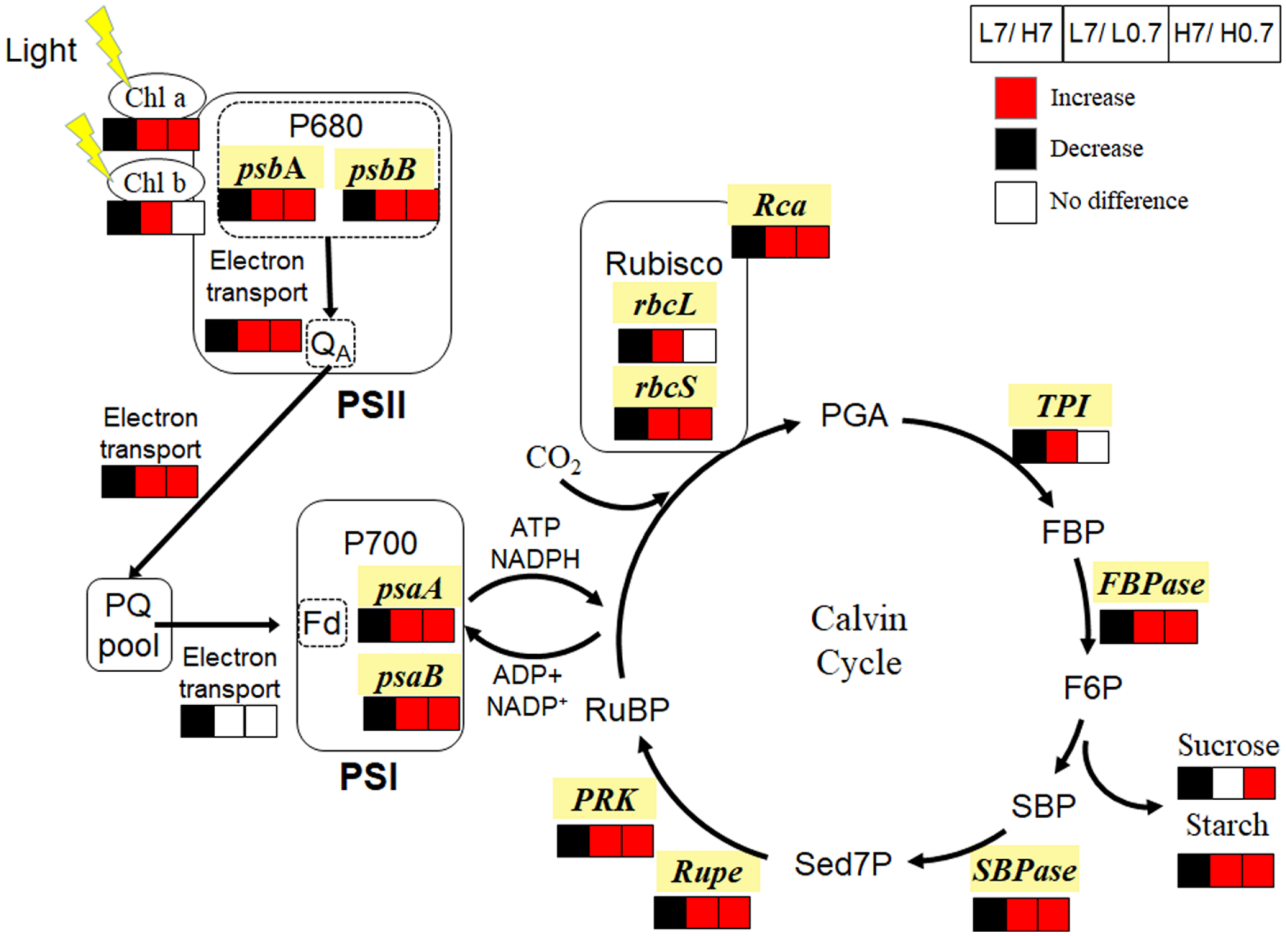
Effect of R/FR on photosynthetic electron transport and Calvin cycle in cucumber leaves under salt stress. Compared to L7 treatment, H7 treatment significantly decreased chlorophyll *a*, chlorophyll *b*, sucrose and starch contents, inhibited electron transport from PSII to PSI, and down-regulated expressions of genes (e.g., *psbA*, *psbB*, *psaA*, *psaB*, *rbcL*, *rbcS*, *Rca*, *TPI*, *FBPase*, *SBPase*, *Rupe* and *PRK*) involved in photosynthesis; While L0.7 treatment significantly increased chlorophyll *a*, chlorophyll *b* and starch contents, improved electron transport from PSII to PQ pool, and up-regulated expressions of genes (e.g., *psbA*, *psbB*, *psaA*, *psaB*, *rbcL*, *rbcS*, *Rca*, *TPI*, *FBPase*, *SBPase*, *Rupe* and *PRK*) involved in photosynthesis. Compared to H7 treatment, H0.7 treatment significantly enhanced chlorophyll *a*, sucrose and starch contents, promoted electron transport from PSII to PQ pool, and up-regulated expressions of genes (e.g., *psbA*, *psbB*, *psaA*, *psaB*, *rbcS*, *Rca*, *FBPase*, *SBPase*, *Rupe* and *PRK*) involved in photosynthesis. The red and black units represent enhancement and inhibition of photosynthesis apparatus or gene expression level, respectively, while white units represent no difference. Chl, chlorophyll; PSII, Photosystem II; P680, primary electron donor of PSII; Q_A_, primary electron acceptor quinone molecule; PSI, Photosystem I; P700, primary electron Chl donor of PSI; PQ pool, plastoquinone pool; Rubisco, Ribulose-1,5-bisphosphate carboxylase-oxygenase; PGA, phosphoglycerate; FBP, fructose-1,6-bisphosphate; F6P, fructose-6-phosphate; SBP, Sedoheptulose-1, 7-bisphosphate; Sed7P, Sedoheptulose-7-bisphosphate; RuBP, ribulose-1,5-bisphosphate.

Compared to L7 treatment, chlorophyll *a* and *b* contents, most JIP-test parameters, expressions of genes (e.g., *psbA*, *psbB*, *psaA* and *psaB*) encoding Photosystem II and I (PSII and PSI) reaction center proteins were significantly reduced by H7 treatment, indicating that salt stress delayed reduction of primary electron acceptor quinone molecule in PSII (Q_A_), and electron transfer from Q_A_^—^ to PQ pool until end acceptors (e.g., Fd, NADP) at PSI electron acceptor side, thus reduced photosynthetic electron transfer capacity (**Figure 7**); Meanwhile, expression levels of all key genes (e.g., *rbcL*, *rbcS*, *Rca*, *TPI*, *FBPase*, *SBPase*, *Rupe* and *PRK*) involved in Calvin cycle were statistically down-regulated, and Rubisco activity, sucrose and starch contents were significantly decreased by H7 treatments. These indicated that salt stress inhibited leaf photosynthesis mainly through disturbing photosynthetic electron transport and Calvin cycle.

L0.7 treatment had the highest chlorophyll *a* and *b* contents, *φ*_Eo_, PI_ABS_, *psbA*, *psbB*, *psaA* and *psaB* gene expressions in all treatments, suggesting that low R/FR accelerated electron transfer from Q_A_^—^ to PQ pool, thus enhanced photosynthetic electron transport capacity; In the meanwhile, expression levels of all key genes (e.g., *rbcL*, *rbcS*, *Rca*, *TPI*, *FBPase*, *SBPase*, *Rupe* and *PRK*) involved in Calvin cycle, and Rubisco activity were highest in L0.7 treatment, contributing to accelerated Calvin cycle and increased starch content in cucumber leaf. In all, low R/FR enhanced leaf photosynthesis mainly through promoting photosynthetic electron transport and Calvin cycle.

Compared to H7 treatment, chlorophyll *a* content, *φ*_Po_, *φ*_Eo_, *psbA*, *psbB*, *psaA* and *psaB* gene expressions were significantly increased by H0.7 treatment, showing that low R/FR enhanced linear photosynthetic electron transport from PSII to PQ pool under salt stress; Simultaneously, expression levels of most genes (e.g., *rbcS*, *Rca*, *FBPase*, *SBPase*, *Rupe* and *PRK*) involved in Calvin cycle were up-regulated and Rubisco activity was enhanced by H0.7 treatment, resulting in increased sucrose and starch contents. In brief, low R/FR benefited leaf photosynthesis mainly through improving photosynthetic electron transport and Calvin cycle under salt stress.

## Discussion

Salt stress severely inhibits leaf photosynthesis and plant growth, and it has become a challenge in vegetable production. R/FR, an important light environmental factor, takes an active part in regulating salt tolerance in plants. Recently, great progress has been made in physiological characteristics and molecular mechanism of vegetable crops under salt stress, however, the regulation roles of R/FR on leaf photosynthetic characteristics under salt stress is still largely unclear (Cao et al., 2018; Wang Y. et al., 2021). In this study, we emphasized that low R/FR promoted photosynthetic electron transport chain and Calvin cycle, finally improved leaf photosynthesis in salt-stressed cucumber plant.

### Low R/FR promoted plant growth under salt stress

Growth parameters are common indicators of plant’s response to salt stress. Salt stress inhibits plant tissues and organs growth, delays plant growth and development, and reduces yield and quality in many plant species, such as tomato, lettuce, sorghum and so on (De Lacerda et al., 2005; Shao et al., 2015; Moncada et al., 2020; Wang W. et al., 2021). In the present study, both RGR and total leaf area were lower for H7 and H0.7 treatments than for L7 treatment, indicating that salt stress seriously inhibited cucumber plant growth (**Figure 2**). Low R/FR plays a vital role in regulating plant growth, it not only promotes cell wall extension which leads to increased leaf area, but also enhances leaf photosynthetic capacity, promotes dry matter accumulation and plant growth (Yang et al., 2020; Tan et al., 2022). In this study, H0.7 treatment had higher RGR and total leaf area than H7 treatment, suggesting that low R/FR alleviated the adverse effects of salt stress on plant growth.

### Low R/FR increased leaf photosynthetic capacity in salt-stressed cucumber leaves

Photosynthesis is a crucial process of absorbing light energy and synthesizing inorganic substance into organic compound in plant. Salt stress usually induces the decline in photosynthetic characteristics in horticultural crops, such as *Ziziphus spina-christi* (L.) Willd., *Dianthus superbus* L. and *Coreopsis tinctoria* Nutt. (Ma et al., 2017; Gorai et al., 2019; Jiang et al., 2021). In the current study, *P*_n_, *C*_i_ and *T*_r_ were significantly lower for H7 treatment than for L7 treatment, indicating that salt stress decreased photosynthetic characteristics (**Figure 3A–D**). Salt stress inhibits water absorption, i.e., insufficient photosynthetic raw materials, leading to a reduction in leaf photosynthesis; On the other hand, salt stress-induced ROS (e.g., ^1^O_2_, O_2_^–^, H_2_O_2_ and HO) severely destroys chloroplast structure and metabolism through oxidation of membrane lipids and membrane proteins, thus adversely impacts photosynthetic capacity (Zhang M. et al., 2016). Furthermore, triose phosphate use (TPU) limitation is widely considered to be one of the main limiting factors of light-saturated photosynthetic rate (Long and Bernacchi, 2003). Interestingly, in CO_2_ response curve, CO_2_ assimilation rate plateaued or slightly declined at the higher CO_2_ concentrations in H7 treatment, suggesting that salt stress significantly decreased rate of triose phosphate use, i.e., CO_2_ utilization, leading to the reduction in sucrose and starch contents (**Figure 3E** and **Table 2**).

It is considered that low R/FR can largely increase superoxide dismutase (SOD), peroxidase (POD) and catalase (CAT) activity, effectively alleviate oxidative damage on chloroplast induced by salt stress (Wang Y. et al., 2021). In this study, H0.7 treatment had higher chlorophyll *a* content, gas exchange parameters (e.g., *P*_n_, *C*_i_ and *T*_r_) and carbohydrate (e.g., sucrose and starch) contents than H7 treatment, indicating that low R/FR had a positive effect on leaf photosynthesis in salt-stressed cucumber plants. This can be further proved by low R/FR-induced improved photosynthetic electron transport and Calvin cycle characteristics below.

### Low R/FR enhanced photosynthetic electron transport capacity in salt-stressed cucumber leaves

Although achievements of photosynthetic characteristics under salt stress have been made in various plant species, effects of R/FR on photosynthetic electron transport capacity under salt stress are still largely unclear (Kalaji et al., 2011; Giorio and Sellami, 2021). Salt stress easily causes oxidation of oxygen-evolving complex (OEC), reaction center protein D1 and antenna protein CP47 in PSII, disrupts their structures and inactivates their function, resulting less electrons (Gururani Mayank et al., 2015). Our study found that *F*_v_/*F*_m_, V_J_ and most JIP-test parameters (e.g., *φ*_Po_, *φ*_Eo_, *φ*_Ro_, RC/ABS, PI_ABS_ and PI_total_) were significantly lower for H7 treatment than for L7 treatment (**Figure 4** and **Table S1**). These results indicated that salt stress suppressed the reduction of Q_A_ in PSII, and electron transfer from Q_A_^-^, through PQ pool, to end acceptors (e.g., Fd, NADP) at PSI electron acceptor side, i.e., salt stress severely inhibited linear photosynthetic electron transport chain from PSII to PSI. In addition, salt stress largely down-regulated expression levels of *psbA* (encoding D1 protein in PSII), *psbB* (encoding CP47 protein in PSII), *psaA* and *psaB* (coding P700 core protein in PSI) genes, delayed the repair rate of PSII and decreased PSI activity, showing that salt stress played adverse roles in electron transport (**Figure 6**).

Studies have shown that R/FR could improve antioxidant enzyme activities, effectively relieve oxidative damage of salt stress, delay decomposition rate of proteins, eventually enhance leaf photosynthesis in plants (Cao et al., 2018). Our study clearly indicated that *F*_v_/*F*_m_, *V*_J_ in OJIP curve, *φ*_Po_ and *φ*_Eo_ were significantly increased by H0.7 treatment when compared to H7 treatment, suggesting that low R/FR effectively increased electron transport from primary electron acceptor to PQ pool through Q_A_. Furthermore, owing to up-regulated expression levels of *psbA*, *psbB*, *psaA* and *psaB* genes, the synthesis rate and efficiency of PSII and PSI were also improved by low R/FR (**Figure 6**). These results indicated that low R/FR improved the structure and function of PSII and PSI under salt stress, thus benefited photosynthetic electron transport under stress. Moreover, low R/FR also generated abundant ATP and NADPH for Calvin cycle by exhibiting strong electron transport capacity.

### Low R/FR accelerated Calvin cycle in salt-stressed cucumber leaves

The Calvin cycle is an important component of carbon assimilation in photosynthesis. Rubisco is the key rate-limiting enzyme in Calvin cycle (Sharwood, 2017). The *rbcL*, *rbcS*, and *Rca* genes encode for large subunit, small subunit and activase of Rubisco, respectively. Previous studies found that salt stress can decrease Rubisco activity (Lin et al., 2018), which is consistent with the phenomenon observed in the current study. Under salt stress, total Rubisco activity, expression levels of *rbcL*, *rbcS*, and *Rca* genes and most key genes (*TPI*, *FBPase*, *SBPase*, *Rupe* and *PRK*) involved in Calvin cycle were remarkedly reduced (**Figures 5** **, 6**). The decline in expression and catalytic activity of Rubisco enzyme and other key enzymes involved in Calvin cycle severely restricts the ability of carbon assimilation (Sobhanian et al., 2010).

The previous studies showed that Low R/FR could enhance leaf photosynthesis by promoting carbon assimilation (Tan et al., 2022). Zhou et al. (2021) have suggested that low R/FR improved leaf photosynthetic capacity by up-regulating *RBCS* (encoding for small subunit of Rubisco) gene expression under calcium nitrate stress. In this study, low R/FR increased total Rubisco activity and up-regulated expression of most key genes (*rbcS*, *Rca*, *FBPase*, *SBPase*, *Rupe* and *PRK*) involved in Calvin cycle. These observations were consistent with the observations in tomato (Zhou et al., 2021) and spinach (Tan et al., 2022). These changes illustrating that low R/FR promoted carbon assimilation under salt stress through upregulating the expression levels and activity of Rubisco and other Calvin cycle enzymes.

## Conclusion

Salt stress severely reduced leaf chlorophyll content, inhibited leaf photosynthesis and delayed plant growth. However, Low R/FR largely alleviated the adverse effects of salt stress on photosynthesis, it not only effectively improved linear photosynthetic electron transport from PSII to PQ pool, but also increased Rubisco activity and accelerated Calvin cycle, improved leaf photosynthesis and accelerated plant growth. In conclusion, low R/FR enhanced leaf photosynthesis by improving photosynthetic electron transport and Calvin cycle in salt-stressed cucumber plants. Therefore, usage of low red to far-red light ratio under salt stress condition could be advantageous for vegetable production.

## Data availability statement

The original contributions presented in the study are included in the article/**Supplementary Materials**. Further inquiries can be directed to the corresponding author.

## Author contributions

YM and XG performed experimental design, experimentation, data collection and analysis, manuscript preparation. BL contributed to project leadership, funding. WW performed experimental design and data analysis. LB revised and edited the manuscript. All authors contributed to the article and approved the submitted version.
