# Supplementary material for: Low red to far-red light ratio promotes salt tolerance by improving leaf photosynthetic capacity in cucumber
Source: Front Plant Sci. 2023 Jan 6;13:1053780. doi: 10.3389/fpls.2022.1053780 (PMC9853560; doi:10.3389/fpls.2022.1053780)
Supplement: Supplementary file 1 [file Table_1.docx]

Supplementary Material

**Supplement Table 1**

| Treatment | F_v_/F_m_ | V_J_ | V_I_ | φP_o_ | φE_o_ | δR_o_ | φR_o_ | RC/ABS | PI_ABS_ | PI_total_ |
| --- | --- | --- | --- | --- | --- | --- | --- | --- | --- | --- |
| L7 | 0.80 ± 0.00 a | 0.51 ± 0.02 bc | 0.8 ± 0.02 a | 0.69 ± 0.02 a | 0.34 ± 0.02 b | 0.42 ± 0.03 a | 0.14 ± 0.02 a | 0.33 ± 0.01 a | 0.76 ± 0.13 b | 0.58 ± 0.14 ab |
| L0.7 | 0.80 ± 0.00 a | 0.62 ± 0.02 a | 0.83 ± 0.03 a | 0.74 ± 0 a | 0.39 ± 0.01 a | 0.39 ± 0.03 a | 0.15 ± 0.01 a | 0.32 ± 0.01 a | 1.03 ± 0.1 a | 0.7 ± 0.11 a |
| H7 | 0.26 ± 0.01 c | 0.48 ± 0.01 c | 0.79 ± 0.02 a | 0.51 ± 0.02 c | 0.19 ± 0.02 d | 0.44 ± 0.05 a | 0.09 ± 0.01 b | 0.27 ± 0.02 b | 0.19 ± 0.04 c | 0.18 ± 0.05 c |
| H0.7 | 0.77 ± 0.01 b | 0.56 ± 0.01 b | 0.8 ± 0.03 a | 0.6 ± 0.02 b | 0.26 ± 0.01 c | 0.45 ± 0.05 a | 0.12 ± 0.02 ab | 0.29 ± 0.01 ab | 0.36 ± 0.05 c | 0.33 ± 0.1 bc |

Note: L7, 0 mM NaCl and R/FR=7; L0.7, 0 mM NaCl and R/FR=0.7; H7, 80 mM NaCl and R/FR=7; H0.7, 80 mM NaCl and R/FR=0.7. Different letters within the same column represent significant differences (P<0.05, n=4).
